# Development of droplet digital PCR-based detection of bacterial pathogens in prosthetic joint infection: a preliminary study using a synthesized model plasmid

**DOI:** 10.3389/fcimb.2023.1301446

**Published:** 2023-11-02

**Authors:** Lee-Jung Tak, Min-Kyoung Shin, Jun-Il Yoo, Min-Chul Cho, Wanil Kim

**Affiliations:** ^1^ Department of Convergence Medical Science, Gyeongsang National University, Jinju, Republic of Korea; ^2^ Department of Microbiology, Department of Convergence Medical Science, and Institute of Health Sciences, School of Medicine, Gyeongsang National University, Jinju, Republic of Korea; ^3^ Department of Orthopedic Surgery, Inha University Hospital, Incheon, Republic of Korea; ^4^ Departments of Laboratory Medicine, Korea University Guro Hospital, Korea University College of Medicine, Seoul, Republic of Korea; ^5^ Department of Biochemistry, Department of Convergence Medical Science, and Institute of Health Sciences, School of Medicine, Gyeongsang National University, Jinju, Republic of Korea

**Keywords:** ddPCR, periprosthetic joint infection, diagnosis, bacteria, infection

## Abstract

Periprosthetic joint infection (PJI) can be diagnosed to characterize the microorganisms constituting a biofilm, which is an essential procedure for proper treatment. The gold standard method for detecting and identifying the causative microorganism is culture of microorganisms from patients-derived sample.; however, this method takes a long time and has low sensitivity. To compensate for these limitations, identification methods based on real-time PCR (RT-PCR) have been widely used. However, RT-PCR also has limitations, including low sensitivity and the requirement of a standard curve for quantification. Therefore, to prevent significant proliferation of pathogenic bacteria, it is important to detect a limited number of infectious bacteria during early stages of PJI. In the present study, we developed droplet digital PCR-based detection of bacterial pathogens in PJI. And we evaluated the analytical performance of the assay using a model plasmid, based on the 16S ribosomal DNA sequence of target bacteria commonly found in PJI. We also prepared genomic DNA extracted from *E. coli*, *S. aureus*, and *S. epidermidis* to test whether ddPCR provides better sensitivity and quantification of the target sequences. ddPCR detected 400 attograms of target DNA, which was more than 10 times less than that detected by real-time PCR using synthesized plasmid. In addition, ddPCR detected target regions from genomic DNA of 50 femtograms for *E. coli*, 70 femtograms for *S. epidermidis*, and 90 femtograms for *S. aureus*. The results indicate that ddPCR has the potential to decrease the microbial detection limit and provide precise detection, signifying its effectiveness for early PJI.

## Introduction

The prevalence of artificial joint transplantation is increasing owing to increased life expectancy and changing lifestyles among the older population (Kurtz et al., 2007). Periprosthetic joint infections (PJI) result in inflammation of the synovial membrane and bone following artificial joint replacement surgery. The number of publications on periprosthetic joint infection continues to grow as the number of PJIs and transplants increases (Li et al., 2020). PJI has been reported to occur in 1%–2% of primary arthroplasties and 4% of revision surgeries (Ong et al., 2009; Aggarwal et al., 2013). PJI is a devastating complication associated with high morbidity rates, prolonged hospitalization, and the need for additional surgery with antimicrobial treatment (Schwarz et al., 2019; Pannu et al., 2021).

PJI can occur either in the early post-implantation phase, typically within the first 4 weeks, or later, usually between 3 months and 3 years after implantation. Early infections are caused by highly virulent pathogens, such as *Staphylococcus aureus*, *Streptococci*, and *Enterococci*, while delayed infections are caused by less virulent organisms (Izakovicova et al., 2019; Gatti et al., 2022). There are many microorganisms causing PJI, such as *Staphylococcus aureus*, coagulase-negative *Staphylococcus*, *Streptococcus* species, Enterococcus species, and gram-negative bacteria (Choong et al., 2007; Kuiper et al., 2014; Patel, 2023). Pathogenic microorganisms adhere to the implants and form microcolonies and biofilms via cell proliferation and intercellular adhesion (Parvizi et al., 2011). The most common bacteria responsible for the formation of such biofilms are *Staphylococcus aureus* and *Staphylococcus epidermidis* (Choong et al., 2007).

Diagnosis of PJI can be performed in multiple steps, including laboratory testing, imaging, and joint aspiration (Izakovicova et al., 2019). The 2018 Evidence-Based Stepwise Algorithm for Diagnosis of PJI is a clinical decision-making tool that provides a systematic approach to the diagnosis of PJI. The algorithm is based on the 2018 Musculoskeletal Infection Society (MSIS) criteria for the diagnosis of PJI, which are the most widely accepted diagnostic criteria for PJI. The algorithm consists of four steps: 1) clinical evaluation, 2) laboratory testing, 3) Imaging studies, 4) Joint aspiration and culture. The 2018 Evidence-Based Stepwise Algorithm for Diagnosis of PJI is a valuable tool for clinicians who are diagnosing PJI. The algorithm provides a systematic approach to the diagnosis of PJI, which can help to improve the accuracy of diagnosis and the quality of care for patients with PJI (Parvizi et al., 2011; Osmon et al., 2013).

Because the identification of microbes is essential for the appropriate treatment of PJI, PJI can be further diagnosed through the detection of pathogenic microbes in the affected tissue, synovial aspirate, and blood. However, the limitations of the detection of microbes in fluids include a high detection limit, difficulty in initial diagnosis, and lack of a method for the simultaneous detection of various pathogenic microbes. The primary and most important method for detecting the causative microorganism is the direct culture of patient-derived samples. Since the introduction of sonication culture, the sensitivity of PJI diagnosis has drastically increased (Rodriguez-Merchan, 2022). However, culturing tissue samples obtained during joint aspiration (i.e., synovial fluid) or surgery remains time-consuming and has low sensitivity. Culture-negative results have been observed in numerous PJI cases, leading to unnecessary antibiotic use or even unnecessary surgery (Berbari et al., 2007; Trampuz et al., 2007; Bellova et al., 2019). If culture-negative PJI are still clinically suspected, a presumptive diagnosis is made using other indirect markers, such as C-reactive protein (CRP), erythrocyte sedimentation rate (ESR), leukocyte esterase, and alpha-defensin (Palan et al., 2019).

Through broad-range PCR method, it might be possible to roughly confirm whether the cause of PJI is a bacterium or fungus prior to accurate bacterial identification of the species. The differential detection of these bacterial and fungal infections provides important information for clinicians in selecting appropriate drugs and determining treatment directions.

Among the PCR-based molecular diagnostic methods, real-time PCR has been widely used instead of traditional PCR. Because this method quantifies the amount of DNA via the cycle threshold (Ct) value, which is defined as the number of cycles for the amplicon-derived fluorescence to exceed the background, a standard curve should be generated for quantitative analyses, which makes this method non-preferred (Kralik and Ricchi, 2017). Significant deviations could also occur in the results owing to differences in many variables, such as amplification efficiency, template processing, and machine error in each trial.

Recently, droplet digital PCR (ddPCR) was introduced for clinical diagnostics. ddPCR divides a mixed volume of polymerase, primers, and templates into tens of thousands of droplets, so that the number of target amplifications can be counted in a digital-like on-and-off manner (Hindson et al., 2011). This method enables absolute quantification of the targets without standard curve generation, as well as quantification of a small number of targets with better sensitivity and accuracy than conventional diagnostic tools (Hindson et al., 2011; Pinheiro et al., 2012). Thus, ddPCR has garnered significant interest in the clinical field, particularly in cases with limited access to *in vivo* samples with mutated genes in hemato-oncology and infectious disease pathogens (Miotke et al., 2014; Li et al., 2018). However, the use of ddPCR for detecting PJI has not yet been reported. Here, we suggest the potential application of ddPCR for diagnosing significant pathogenic microbes with high sensitivity and accuracy, so that the method could be used in the clinical determination of PJI.

## Materials and methods

### Determination of target region and primers

The primers and probes targeting common sequences on 16S rRNA of PJI significant microbes are described in previous study (Horz et al., 2005). Briefly, a universal primer sequence was determined using the ‘Probe Match’ of ARB phylogenetic software, a database for maintaining and managing sequence data. The universal PCR primer and probe sequences were determined through in silico 16S ribosomal RNA gene sequence analysis of 43 sub-strains of *S. aureus*, 7 sub-strains of *S. epidermidis*, and 4 sub-strains of *E. coli*. Common 16S ribosomal RNA regions for targeting were selected from reference genomes of *Escherichia coli* (Genbank ID: MF.372553.1), *Staphylococcus aureus* (Genbank ID: MN524176.1), and *Staphylococcus epidermidis* (Genbank ID: OP481211.1).

### Plasmid DNA transformation and Midi prep

A model plasmid was synthesized based on the 16S ribosomal DNA sequences of target bacteria commonly present in PJI. The target DNA fragment was inserted into pUCosmo-Amp provided by Cosmogenetech (Cosmogenetech, Daejeon, Korea). 1µl of plasmid DNA was added to DH5α chemically competent *E. coli* (Enzynomics, Daejeon, Korea), followed by inoculation on an ampicillin selection plate and incubated overnight at 37°C. Midi prep of the plasmids was performed with 250 ml culture of the transformed bacteria, and DNA was then obtained using the NucleoBond^®^ Xtra Midi Plus kit (MACHEREY-NAGEL, Düren, NW, Germany). DNA quantification was measured at 260 nm using a QIAxpert spectrophotometer (QIAGEN, Hilden, Germany).

### Design of primers and probes

Primers and probes were designed using the 16S ribosomal RNA sequences inserted into plasmid DNA and are shown in the below. FAM was selected as the receptor dye of the probe, and BHQ-1 was chosen as the quencher dye.

Forward primer: 5`-TCCTACGGGAGGCAGCAGT-3`

Reverse primer: 5`-GGACTACCAGGGTATCTAATCCTGTT-3`

Probe: 5`-[Fam]CGTATTACCGCGGCTGCTGGCAC[BHQ-1]-3`

### Qualitative conventional PCR

Template DNA was continuously diluted 10 times to a concentration of 400ag. A sample containing primers only was used as a negative control. PCR was performed using a Go-Taq (Promega, Madison, WI, USA) and a VeritiPro thermal cycler (Thermo Fisher scientific Inc., Waltham, MA, USA). The amplification program was one cycle for 5 minutes (95°C), followed by 30 cycles of 30 seconds at 95°C, 30 seconds at 55°C, and 30 seconds at 72°C. The amplicon was also visualized under gel electrophoresis for size control. Electrophoresis was performed at 50 volts using 2% agarose gel (FMC bioproduct, Philadelphia, Pennsylvania, USA). The agarose gel was stained and visualized using MaXidoc Gel Imaging System (DAIHAN scientific, Wonju, Korea).

### Real-time PCR

Real-time PCR was performed with PCR Master Mix (GenDEPOT, Barker, Texas, USA) using the Rotor-Gene Q device (QIAGEN, Hilden, Germany). Samples were diluted in the same manner as qualitative PCR. Amplification was performed for 5 minutes at 95°C for activation, followed by 30 cycles of 30 seconds at 95°C, 30 seconds at 55°C, and 30 seconds at 72°C. CT values were plotted using Graphpad Prism software (Graphpad Software Inc, San Diego, CA, USA).

### Droplet-digital PCR

Target DNA was also quantified using a QX200 droplet digital PCR instrument (Bio-Rad, Hercules, CA, USA). We prepared 20μl of ddPCR reaction mix containing ddPCR Supermix for Probes (no dUTP) (Bio-Rad, Hercules, CA, USA), DNA template, and primer/probe. A 40 μL emulsified mixture was prepared by combining 20 μL PCR mix and probe droplet formation oil (Bio-Rad, Hercules, CA, USA). All the procedures were performed on a QX200 Droplet Digital PCR System (Bio-Rad, Hercules, CA, USA). After droplet generation, the emulsified mixture was transferred to a clean 96-well plate and sealed with the PX1 PCR plate sealer (Bio-Rad, Hercules, CA, USA) at 180°C for 5 seconds. The emulsified mixture was then PCR amplified. The VeritiPro Thermal Cycler equipment was used for amplification (Thermo Fisher Scientific Inc., Waltham, MA, USA). The thermal cycling involved 40 cycles of 30 seconds at 94°C and 1 minute at 60°C, followed by a 10-minute incubation at 95°C to terminate the PCR reaction. The instrument had a ramp rate of 2°C/sec for all steps. QuantaSoft was used to determine the number of positive droplets (Bio-Rad, Hercules, CA, USA).

### Bacteria genomic DNA extraction

*Escherichia coli* (*E. coli* KBN12P06660), *Staphylococcus aureus* (*S. aureus* KBN12P06533), and *Staphylococcus epidermidis* (*S. epidermidis* KBN12P06690) were provided from the Fastidious Specialized Pathogen Resources Bank (a member of the National Culture Collection for Pathogens), Gyeongsang National University Hospital, Jinju, Korea. All bacteria strains used in the study were obtained from clinical samples. Bacterial DNA was extracted using PureLink™ Genomic DNA Mini Kit (Thermo Fisher scientific Inc., Waltham, MA, USA) according to the manufacturer’s instruction.

## Results

### Determination of primer design and target

**Figure 1:**
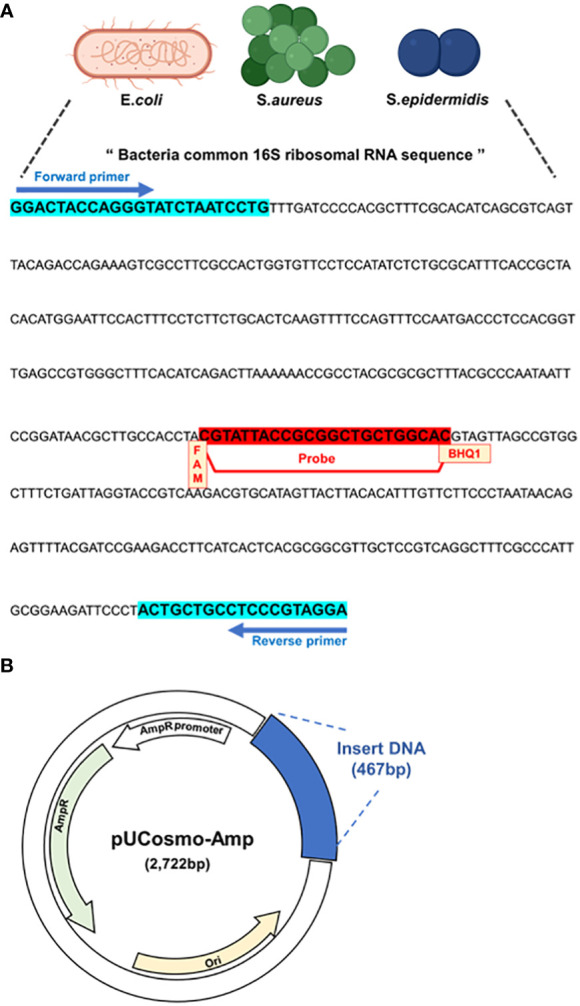
Model plasmid and primer design. **(A)** Primers and probe that target common sequences on 16S rRNA of PJI significant bacteria, *E coli*, S. *aureus* and S. *epidermidis*. The probe has FAM as the receptor dye and BHQ-1 as the quencher dye. **(B)** Schematic of model plasmid. The target DNA fragment was inserted into ampicillin-resistant pUCosmo-Amp plasmid provided by Cosmogentech. The plasmid was then added to DH5α chemically competent *E coli*, transformed, and then used in the experiment.

We utilized a common 16S rRNA sequence from a previous study (Horz et al., 2005) and conducted all the analyses in this study. This sequence included the common 16S rRNA of *E. coli*, *S. aureus*, and *S. epidermidis*, which are the most frequent and representative pathogenic bacteria detected in PJI (**Figure 1A**). Primers and probes were designed and based on this sequence, along with the FAM dye and BHQ-1 quencher. Next, a model plasmid was created by synthesizing the target sequence and inserting it into a simple plasmid (**Figure 1B**).

### Construction of synthesized 16s rRNA sequence inserted plasmid for the analysis of limit of detection

**Figure 2:**
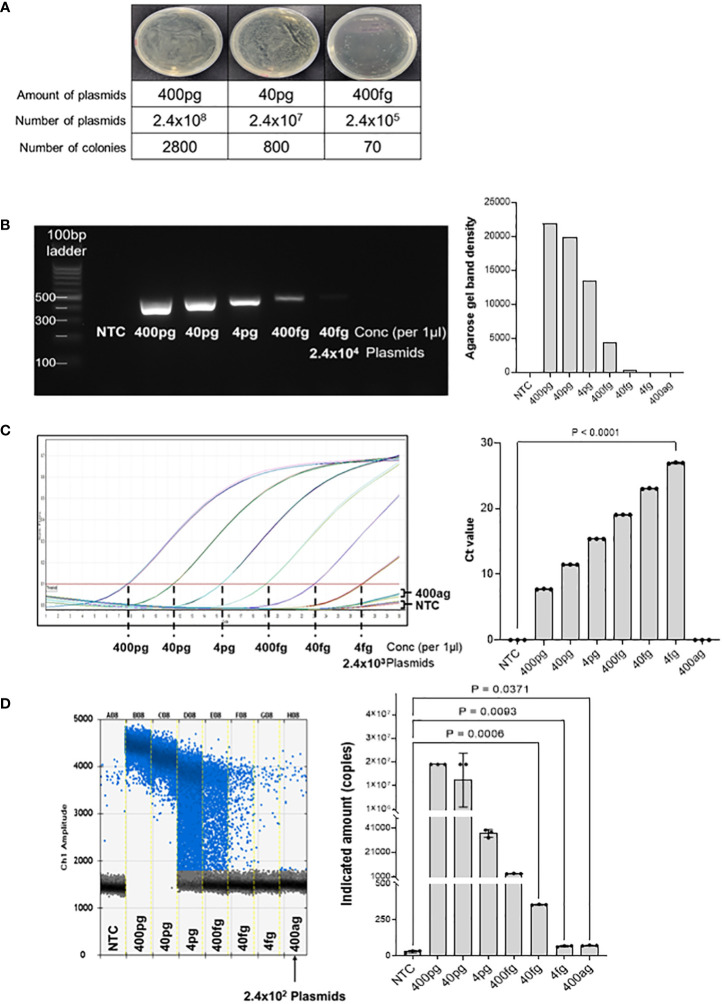
Threshold determination with model plasmid. **(A)** The model plasmid containing the target sequences was serially diluted and transformed into *E coli* to quantify the number of colonies. The results show that 400 fg of model plasmid was the minimum amount to detect visible colonies on the plate. The number of colonies was quantified using Image J program. **(B)** The model plasmid was PCR-amplified using Taq polymerase and visualized by electrophoresis on a 2% agarose gel. As the concentration decreased, the band density also decreased. There was no band on the no template control and concentration below 400 fg. **(C)** Quantitative real-time PCR was performed for 30 cycles to determine the Ct (cycle threshold) of the diluted model plasmid. The results were visualized using GraphPad software. 400 fg of DNA reached a plateau at the end of the cycles, which was significantly different from the control. 4 fg of DNA did not reach a plateau for Ct analysis compared to the control, but was visually different from the control. **(D)** Digital droplet PCR was performed to determine the detection limit of the diluted plasmids. The raining drops between 4 pg and 40 fg range could be optimized, but since the purpose of this method is to detect any bacteria in the samples qualitatively, this would not be a significant flaw of this method. The LOD of the ddPCR-based detection was 100 pg, corresponding to 2.4x10^2^ plasmids. The fluorescence value generated by DNA amplification was assessed as the indicated amount, and positive droplets containing target DNA were counted using a droplet reader and displayed in blue. The measured positive droplet was converted to a calculated value according to Poisson’s Law of Dispersion in the analysis program and displayed in the graph. Results are shown as mean ± SD, one-way ANOVA test. NTC, no template control; pg, picogram; fg, femtogram; ag, attogram; conc, concentration.

Ampicillin-resistant pUCosmo-Amp™ provided by Cosmogenetech was used as the vector, and purified DNA was serially diluted to determine the minimum detection threshold. To reduce the time required for the extraction of genomic DNA from microorganisms and compensate for the purity and low yield of the final product owing to the many intermediate steps, we analyzed the detection of common 16S sequences based on the synthesized model plasmids. The number of transformed colonies decreased as the DNA was diluted (**Figure 2A**). 400 ng of the plasmid DNA was serially diluted and transformed into DH5α *E. coli*, and the colony number was quantified. Transformation with 400 fg of the plasmid (2.4x10^5^ plasmids) yielded 70 colonies; however, we could not find any colonies under 400 fg.

### Test using conventional PCR

Next, we PCR-amplified the diluted DNA to assess the minimal number of plasmids with a detectable signal on agarose gel electrophoresis (**Figure 2B**). DNA of the concentration of 400 pg was 1/10 diluted and amplified using Taq polymerase, followed by visualization on a 2% agarose gel. The result shows that 400 fg of DNA (2.4 × 10^5^ plasmids) was the minimum detectable concentration, which is consistent with the transformation analysis in **Figure 2A**. Band quantification using ImageJ also decreased as the DNA diluted. Therefore, there was no difference in the detection limit between the PCR and bacterial transformation methods for the target 16S rRNA sequence.

### Analysis of limit of detection using real-time PCR

Next, we performed quantitative real-time PCR to assess the detection limit of the diluted plasmids (**Figure 2C**). As the DNA concentration decreased, the threshold cycle number (Ct) increased, and there was no significant difference between the no-template control and the 400 ag (attogram) DNA. When 40 fg or less of DNA was used for the analysis, a plateau was not reached, but there was a clear graphical difference from the no-template control (NTC). However, the amplification curve for 4fg DNA was not significantly different from that of NTC. Therefore, we assume the minimum amount of target DNA that can be detected by real-time PCR to 40 fg or more.

### Analysis of LOD using ddPCR

Subsequently, the detection threshold was determined by ddPCR analyses with serially diluted DNA (**Figure 2D**). We found that 400 ag of the plasmid DNA (240 plasmid copies) generated an average of 74 positive droplets. However, the difference in the number of droplets between 4fg and 400ag was insignificant. Therefore, we concluded that the ddPCR assay could detect 4fg, which corresponds to 2400 copies of target sequence fragments. Taken together, these results suggest that the detection of target sequences through ddPCR is advantageous for quantitative analysis compared to conventional real-time PCR analysis and has the potential to lower the minimum detection limit.

### Comparison of LOD of real-time PCR with that of ddPCR using bacterial genomic DNA

**Figure 3:**
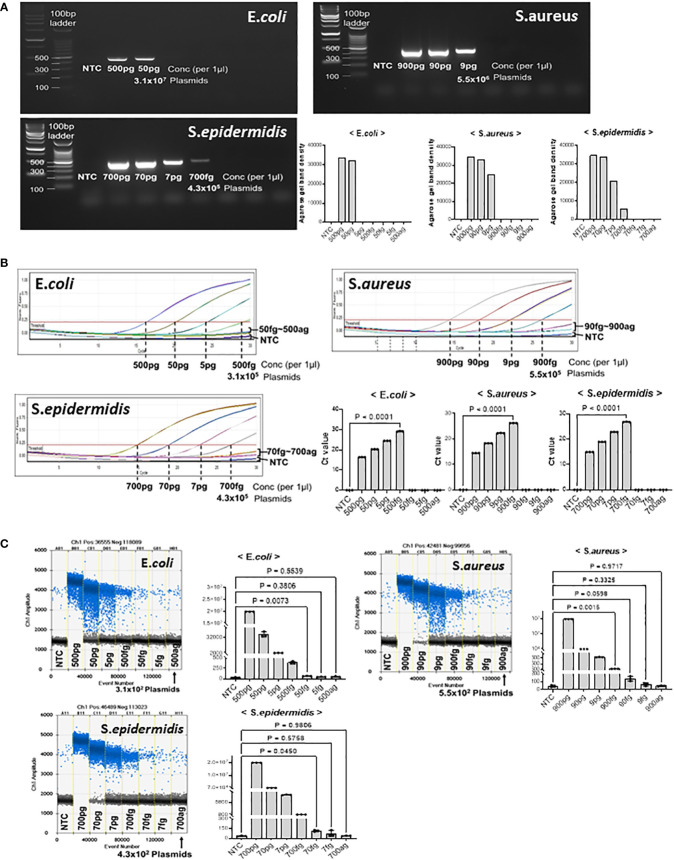
Threshold determination with genomic DNA from *E coli*, *S. aureus*, and *S. epidermidis*. PJI-significant bacteria were cultured and collected for genomic DNA purification. Genomic DNA from each bacterium was then serially diluted to determine if our method could amplify and detect target sequences with genomic DNA from cultured bacteria. **(A)** Total genomic DNA was diluted and PCR amplified. The LOD for *E coli* was 50 pg, the LOD for *S. aureus* was 9 pg, and the LOD for *S. epidermidis* was 700 fg. The LOD of the bacteria showed large difference with the same primer sets, which could be due to the different complexity and obstacle around the target sequences. The results were visualized on a 2% agarose gel. The band density was quantified by Image J program. **(B)** Real-time PCR was performed to determine the Ct of bacterial genomic DNA. We determined the LOD even if the DNA amplification did not reach a plateau, if there was a visual difference between the control and the sample on the graph. The real-time PCR analysis for all three bacteria showed significant differences in genomic DNA at the level of hundreds of fg compared to the control. **(C)** ddPCR was performed to determine the LOD with genomic DNA from PJI significant bacteria. For *S. aureus*, ddPCR showed the same LOD as real-time PCR, but for *E coli* and *S. epidermidis*, it was 10 times more sensitive than real-time PCR, detecting 50 fg and 70 fg, respectively. The positive and negative droplets as classified by the thresholds are shown in blue and grey, respectively. The calculated value of the positive droplet according to Poisson’s law of dispersion was graphed. Results are shown as mean ± SD, one-way ANOVA test. NTC, no template control; pg, picogram; fg, femtogram; ag, attogram; conc, concentration.

We tested the detection limits of gDNA extracted from *E. coli*, *S. aureus*, and *S. epidermidis* at different dilutions. First, we confirmed the detection limit of the PCR-amplified diluted DNA by agarose gel electrophoresis (**Figure 3A**). The minimum detectable concentrations were 50 pg in *E. coli*, 9 pg in *S. aureus*, and 700 fg *S. epidermidis*. Band quantification using ImageJ also showed a decrease in band intensity with DNA dilution. In the PCR amplification results, the detection limits for *E. coli* and *S. aureus* were higher than those in our model plasmid experiment, whereas the detection limits for *S. epidermidis* were confirmed to be similar.

Quantitative real-time PCR was performed to confirm the detection limit of the diluted microorganisms (**Figure 3B**). As the concentration of the microorganisms decreased, the Ct value increased. Significant differences were observed between the no-template control and *E. coli* at 500 fg, *S.aureus* at 900, and *S. epidermidis* at 700 fg. The DNA amplification curves of less than 50 fg for *E. coli*, 90 fg for *S. aureus*, and 70 fg for *S. epidermidis* were not significantly different from those of NTC, nor were the Ct values. Therefore, we suggest that the minimum amount of microbial DNA detected by real-time PCR is 500 fg in *E. coli*, 900 fg in *S. aureus*, and 700 fg in *S. epidermidis*.

Considering that ddPCR can efficiently reduce the detection limit in model plasmid experiments, we performed ddPCR analysis with serially diluted microorganism DNA (**Figure 3C**). In *E. coli*, 50 fg of plasmid DNA (31,000 plasmid copies) generated an average of 66 positive droplets. However, the number of droplets generated at < 5 fg was negligible. Next, in *S. aureus*, 900 fg of plasmid DNA (5.5 x10^5^ plasmid copies) generated an average of 1246 positive droplets. Compared to 90–900 ag (attogram), 900 fg of *S. aureus* DNA generated significantly more positive droplets than the no-template control. We found that *S. epidermidis* DNA generated an average of 114 positive droplets (43,000 plasmid copies) at a concentration of 70 fg. However, the number of droplets generated at 7 fg and 700 ag was insignificant. Thus, the limit of detection (LOD) of ddPCR was determined to be 50 fg for *E. coli*, 900 fg for *S. aureus*, and 70 fg for *S. epidermidis*.

## Discussion

One prominent method for diagnosing PJI involves detecting infectious bacteria by real-time PCR. Among the microbes that cause PJI, bacteria account for over 97% of the cases, with fungi accounting for the remaining cases (Benito et al., 2016). In bacteria, there is a common region suitable for universal amplification of 16rRNA (Plouzeau et al., 2015). Similarly, in fungi, conserved regions within the 18S, 5.8S, and 28S ribosomal subunits can be targeted for universal amplification (Petti, 2007). Thus, the application of broad-range PCR, which can be amplified and detected by targeting the common regions of these bacteria, is feasible (Bemer et al., 2014).

In this study, we confirmed that the designed primer set and probe targeting the 16S ribosomal RNA sequences worked properly in tests using conventional PCR and real-time PCR prior to the main ddPCR experiment. The melting temperatures of the primers and probes were optimized using conventional real-time PCR. To facilitate the limit-of-detection analysis, we constructed a plasmid containing an artificially synthesized 16s rRNA sequence. In experiments using this plasmid, the LOD of real-time PCR and ddPCR were confirmed to be 40 fg and 4 fg, respectively, confirming that the LOD of ddCPR was approximately 10 times lower than that of real-time PCR. In addition, to perform LOD analysis in a situation similar to an actual clinical situation, we extracted and tested the gDNA of *S. aureus, S. epidermidis, and E. coli*, which have been reported as the main causative bacteria of PJI. In the LOD experiment using bacterial gDNA, the LOD differed slightly for each strain. The LODs of real-time PCR was 500 fg for *E. coli*, 900 fg for *S. aureus*, and 700 fg for *S. epidermidis*, whereas the LODs of ddPCR was 50 fg for *E. coli*, 900fg for *S. aureus*, and 70 fg for *S. epdermidis* confirming that the LOD of ddPCR was approximately 10/1 compared to real-time PCR.

Although culture is still the gold standard for diagnosis of PJI, in order to increase sensitivity and diagnose PJI as quickly as possible, molecular diagnosis based on real-time PCR has recently been widely used as an auxiliary tool for diagnosis of PJI (Rougemont et al., 2004). However, these real-time PCR-based molecular diagnostic methods do not show satisfactory results in terms of sensitivity, accuracy, or replicability when the concentration of the infectious agent is low during the early stages of infection (Li et al., 2018). The ddPCR technique used in our study has the advantage of being more sensitive than real-time PCR and enables more accurate quantitative testing without a separate control material. These characteristics further strengthen the possibility of using ddPCR as a molecular diagnostic method for detecting infectious agents in PJI.

Because there is no specific diagnostic method for PJI, diagnosis of PJI is diagnosed by performing many tests and comprehensively interpreting the results. These include laboratory and imaging studies. Laboratory tests included non-specific inflammatory markers, such as serum C-reactive protein (CRP), erythrocyte sedimentation rate (ESR), procalcitonin, peripheral blood leukocytes, synovial fluid (SF) white blood cells, and bacterial cultures of preoperative SFs and intraoperative tissues (Peel et al., 2012; Saleh et al., 2018). However, these systemic inflammatory markers are often normal in PJIs caused by low-virulence pathogens (Dodson et al., 2010; Piper et al., 2010; Perez-Prieto et al., 2017).

According to the PJI treatment guidelines, when PJI is diagnosed, surgery to replace an artificial joint is required; therefore, it is very important to clearly detect the source of infection to determine the direction of treatment (Izakovicova et al., 2019). In addition, bacteria account for approximately 97–99% of PJI infectious agents, and fungi account for approximately 1–3%, and are caused by mycobacteria at a very low frequency (Benito et al., 2016). When referring to the frequency of such PJI infectious agents, confirming the presence of bacteria in specimens such as joint fluid in patients suspected of having PJI would be very useful for determining the treatment strategy. With this rationale, we developed a diagnostic method that focuses on detecting the bacteria that account for the largest proportion of the causes of PJI. Gram-positive bacteria are the main species detected in PJI after joint replacement surgery, but gram-negative bacteria account for a low percentage (Wang et al., 2018). Among Gram-positive bacteria, *Staphylococcus epidermidis* and *Staphylococcus aureus* had the highest frequency, and among Gram-negative bacteria, *Escherichia coli* accounted for the highest frequency (Benito et al., 2016). In our study, the test was conducted using three bacterial strains that accounted for such a high frequency.

In this study, we observed a higher LOD for gDNA samples from cultured bacteria than in purified model plasmid used in other experiments, which could be due to the following reasons. First, some of the target DNA may have been lost during the extraction of genomic DNA from the bacteria. This can be solved in the future by optimizing the extraction process for the appropriate genomic DNA. Second, the target region is surrounded by many non-target regions that may interfere with primer binding. In the future, it may be possible to select and detect the target region through treatment with restriction enzymes (frequent cutters). Third, the target DNA sequence must be contained within the ddPCR droplet for proper results; because the bacterial chromosome exists as a continuous macromolecule, physical space limitations may have occurred. This may be addressed in the future by treatment with the appropriate restriction enzymes.

Overall, in *E. coli* and *S. epidermidis*, the results clearly showed that ddPCR is advantageous for quantitative analysis compared to prior real-time PCR analysis. For *S. aureus*, the LOD was 900 fg, which was similar to that of real-time PCR. However, at 90 fg, the average number of ddPCR-positive droplets was 140, which was significantly higher than that of the control (44 droplets). Although it did not show statistical significance in this report, further studies could optimize the ddPCR parameters so that the assessment of samples under 900fg could be possible in the future. In addition, for *S. aureus*, when comparing ddPCR and real-time PCR, the detection limit with an effective value was 900 fg. However, considering that ddPCR had 140 positive droplets at a 90 fg concentration compared with NTC (44 droplets), it is considered to have a clear difference from the no-template control. Previous studies have reported that the LOD of ddPCR is approximately 1000 times lower than that of real-time PCR, but it was confirmed to be approximately 10 times lower in this study. It can be inferred that this difference is probably caused by a problem with the nucleic acid extraction method, such as DNA loss during the nucleic acid extraction process, or because the protocol of our ddPCR method has not yet been fully optimized. Thus, for future clinical applications of ddPCR detection technology, validation based on different bacteria involved in PJI infection is required. In addition, the optimization and validation of protocols for handling human-derived samples (such as synovial fluid) and obtaining genomic DNA of sufficient quality for testing are needed. The ddPCR can also identify genetic markers associated with resistance to specific pathogens. According to several reports (McEvoy et al., 2018; Zmrzljak et al., 2021) ddPCR has higher sensitivity to detect somatic mutations, enabling the determination of antibiotic resistance caused by a small number of mutations using specific primer sets.

This study had several limitations. First, the ddPCR method we developed was applied to only three bacteria, and has been applied to more diverse bacteria; therefore, it was not confirmed that all bacteria could be detected by the ddPCR method. Second, the ddPCR method developed in this study was evaluated using synthesized DNA sequences and gDNA of cultured bacteria, and clinical specimens, such as joint fluids of patients, could not be evaluated. In this study, we have presented preliminary data confirming the potential of ddPCR for bacterial detection in PJI and have evaluated its analytical performance. However, additional validation with clinical samples is essential to establish the utility of our ddPCR assay for the diagnosis of PJI in real patients

## Conclusions

In this study, we developed a method for detecting bacteria in PJI using a ddPCR platform, which is known to be more sensitive than real-time PCR. It was confirmed that the method we developed properly worked in plasmids into which artificially synthesized DNA sequences were inserted and in actual Gram-negative and Gram-positive bacteria. In addition, by comparing this method with the real-time PCR method, it was confirmed that it is a more sensitive method with a low LOD of approximately 1/10. Therefore, the ddPCR-based assay we developed is a highly sensitive diagnostic method that can significantly help in detecting bacteria in patients with PJI.

## Data availability statement

The original contributions presented in the study are included in the article/supplementary material. Further inquiries can be directed to the corresponding authors.

## Author contributions

L-JT: Conceptualization, Data curation, Formal Analysis, Investigation, Visualization, Writing – original draft. M-KS: Resources, Supervision, Validation, Writing – review & editing. J-IY: Conceptualization, Supervision, Validation, Writing – review & editing. M-CC: Funding acquisition, Methodology, Supervision, Writing – original draft, Writing – review & editing. WK: Conceptualization, Funding acquisition, Investigation, Project administration, Supervision, Validation, Writing – original draft, Writing – review & editing.
